# Genomic characterization of dengue virus serotype 2 during dengue outbreak and endemics in Hangzhou, Zhejiang (2017–2019)

**DOI:** 10.3389/fmicb.2023.1245416

**Published:** 2023-08-25

**Authors:** Hua Sun, Wenwu Yao, Abubakar Siddique, Fan He, Min Yue

**Affiliations:** ^1^Zhejiang Hospital of Integrated Traditional Chinese and Western Medicine, Hangzhou, China; ^2^Department of Microbiology, Zhejiang Provincial Center for Disease Control and Prevention, Hangzhou, China; ^3^Hainan Institute of Zhejiang University, Sanya, China; ^4^Department of Veterinary Medicine, Institute of Preventive Veterinary Sciences, College of Animal Sciences, Zhejiang University, Hangzhou, China; ^5^State Key Laboratory for Diagnosis and Treatment of Infectious Diseases, National Clinical Research Center for Infectious Diseases, National Medical Center for Infectious Diseases, The First Affiliated Hospital, College of Medicine, Zhejiang University, Hangzhou, China

**Keywords:** dengue virus, arboviruses, whole genome sequencing, phylogenetic analysis, recombinant analysis

## Abstract

**Introduction:**

Dengue fever (DF) is a mosquito-borne viral disease caused by the dengue virus (DENV). In recent years, Hangzhou has undergone a DF epidemic, particularly in 2017, with an outbreak of 1,128 patients. The study aimed to investigate the genetic diversity and molecular evolution among the DF clinical isolates during and after the outbreak to aid in mapping its spread.

**Methods:**

To understand the genetic diversity, 74 DENV-2 strains were isolated from DF epidemic cases between 2017 and 2019. Combining whole genome sequencing (WGS) technology, additional phylogenetic, haplotype, amino acid (AA) substitution, and recombination analyses were performed.

**Results:**

The results revealed that strains from 2017 were closely related to those from Singapore, Malaysia, and Thailand, indicating an imported international transmission. Local strains from 2018 were clustered with those recovered from 2019 and were closely associated with Guangzhou isolates, suggesting a within-country transmission after the significant outbreak in 2017. Compared to DENV-2 virus P14337 (Thailand/0168/1979), a total of 20 AA substitutions were detected. Notably, V431I, T2881I, and K3291T mutations only occurred in indigenous cases from 2017, and A1402T, V1457I, Q2777E, R3189K, and Q3310R mutations were exclusively found in imported cases from 2018 to 2019. The recombination analysis indicated that a total of 14 recombination events were observed.

**Conclusion:**

This study may improve our understanding of DENV transmission in Hangzhou and provide further insight into DENV-2 transmission and the local vaccine choice.

## Introduction

Dengue virus (DENV) is the most prevalent arbovirus, belonging to the Flavivirus genus of the Flaviviridae family, and primarily transmitted by *Aedes aegypti and Aedes albopictus* (Bhatt et al., 2013; Ngwe Tun et al., 2016; Jiang et al., 2018). It comprises four antigenically distinct serotypes (DENV-1, DENV-2, DENV-3, and DENV-4), which are closely related. Dengue fever remains common in tropical and subtropical areas (Kok et al., 2022). There is a global outbreak of DENV, with an estimate of up to 3 million cases reported across more than 180 countries (Brady et al., 2012). According to the World Health Organization (WHO), climate change, economic integration, and migration have contributed to the spread of DENV strains over the last decade (Kim et al., 2015). In China, DF is a significant public health concern, with over 700,000 cases reported in the last 40 years, primarily in the southeastern coastal regions of Guangdong, Hainan, Fujian, Zhejiang, and Taiwan (Almeida et al., 2005; Jiang et al., 2013; Sun et al., 2017).

The genome of DENV is a linear, non-segmented, single-stranded positive-sense RNA (ssRNA+), with approximately 10,700 nucleotides consisting of 5′ and 3′ untranslated regions (UTRs) and an Open Reading Frame (ORF) that encodes 3,391 amino acids. The ORF encodes three structural proteins [Capsid (C), Membrane (prM/M), and Envelope (E)] as well as seven non-structural proteins (NS1, NS2A, NS2B, NS3, NS4A, NS4B, and NS5; Kuhn et al., 2002; Li et al., 2017). DENV was divided into four serotypes based on the antigenicity of the E protein, and each serotype was subdivided into several genotypes based on viral gene sequences (Weaver and Vasilakis, 2009; Lee et al., 2012). Each serotype can cause a diverse range of illnesses in humans, from mild dengue fever (DF) to life-threatening dengue hemorrhagic fever (DHF) and dengue shock syndrome (DSS).

Zhejiang province, with its typical subtropical climate consisting of plains, hills and mountains, offers ideal conditions for mosquito growth and may serve as a potential reservoir for DENV transmission (Guo et al., 2014). An unexpected dengue outbreak in Hangzhou, Zhejiang, resulted in 1,229 cases (the highest number) in a calendar year in 2017. Most of the DENV cases reported in Zhejiang Province were serotype 2, with only a few reported cases of DENV serotypes 1, 3, and 4 (Yan et al., 2018; Yao et al., 2021). According to these reports, DF cases have been reported almost every year in Zhejiang for the past few years. Ongoing surveillance and genomic investigations are critical to assessing potential epidemics in the future. Therefore, this study aimed to investigate the genomic sequences and comprehensive genetic diversity of 74 DENV-2 strains isolated from outbreaks and epidemics during 2017–2019 in Hangzhou.

## Materials and methods

### Ethical statement

The Institutional Ethical Committee of the Zhejiang Provincial Center for Disease Control and Prevention reviewed and approved this human-participant study. According to national legislation and institutional requirements, no written informed consent was required for participation in this study.

### Sample collection

From 2017 to 2019, during the dengue outbreak in Hangzhou, Zhejiang province, serum samples were collected from DENV-positive patients at the Center for Disease Control and Prevention (CDC). The diagnostic criteria established by the Chinese Ministry of Health (WS216-2008) were used to test all DF cases for DENV nucleotide detection and serotype identification.

### Virus isolation

DENV-2 strains isolated from the positive serum of patients in this study were propagated using *Aedes albopictus* gut cell lines (C6/36). The serum samples were diluted tenfold with fresh minimum essential medium (MEM; Gibco, Waltham, United States). 100 μL of diluted serum samples were inoculated with C6/36 cell monolayers and incubated in MEM medium supplemented with 2% fetal bovine serum (FBS, Gibco, United States) at 28°C for 5–7 days. Cells showed typical cytopathic effects (CPE) and were considered DENV-2 positive (Yan et al., 2018). These culture supernatants were then stored at −80°C for use.

### RNA extraction and genome sequencing

The viral RNA extraction was performed as previously described (Spackman and Lee, 2014) from C6/36 cells using the viral RNeasy Mini Kit (Qiagen, Germany) according to the manufacturer’s instructions. Dengue virus RNA was amplified by multiple PCR assays targeting 10 overlapping fragments for covering the genome with Q5 High-Fidelity DNA Polymerase (NEB M0491). The primer sets were presented in a previous study (Cruz et al., 2016). RT-PCR amplified the two overlapping fragments in the virus gene with the following protocol: initial reverse transcription at 50°C for 30 min, and then denaturation at 94°C for 2 min, 40 cycles of denaturation step at 94°C for 30 s, annealing at 53°C for 30 s, primer extension at 72°C for 2 min, and a final extension step at 72°C for 10 min. The nanopore gridion sequencer with flow cell R9.4.1 (FLO-MIN106D) was used to perform dengue virus amplicon sequencing. Guppy v5.0.17 was applied to basecall Q10 above raw reads in super accuracy mode. The purity of PCR products was analyzed by agarose gel electrophoresis. The PCR products were sequenced using NGS by Sangon Biotech Co. (Shanghai, China).

### Genomic characterization and phylogenetic analysis

The sequences were assembled using DNASTAR version 7.0. The assembled nucleotide sequences and translated amino acid sequences were analyzed by BioEdit v7.2. 74 DENV-2 complete genome sequences were isolated during this study, and 166 reference sequences downloaded from the NCBI GenBank database were used to perform phylogenetic analysis. The phylogenetic tree based on complete genome sequences of DENV-2 was constructed using the neighbor-joining method in IQ-TREE, as described previously (Li et al., 2022c; Tang et al., 2023; Wang et al., 2023). The whole genome sequences of 74 strains with translated amino acid (AA) sequences mutations were analyzed by Prokka Toolkit as stated before (Xu et al., 2021; Li et al., 2022a,b), and the secondary structure of structural proteins of DENV-2 epidemic and reference strains were predicted with the RNAfold (Hofacker, 2003; Gruber et al., 2015). Haplotype analyses for the 74 DENV-2 complete genome sequences were conducted using the DNAsp software (Rozas et al., 2017). The sequences were divided into three groups based on the year (DENV2017, DENV2018, and DENV2019), and a haplotype network was created using PopART version 1.7 (Bandelt et al., 1999; Leigh and Bryant, 2015).

### Genetic recombination analysis

Recombination and molecular evolution analyses were conducted with the RDP package (Martin et al., 2021). Potential recombination events across the entire genome and the location of recombination breakpoints and likely parental sequences were identified as previously described (Han et al., 2022).

## Results

### Epidemiological characteristics

All 74 patients reported typical dengue-like symptoms, including vomiting, headache, fever, joint pain, vascular leakage, pleural effusion, myalgia, and nausea. Laboratory investigation results showed low platelet counts (< 100 * 10^9^/L) and elevated liver enzyme levels (>100 U/L), such as alanine aminotransferase and aspartate aminotransferase. Among the 74 DENV-2 strains, 39 (51.3%) were isolated from patients reported in 2017, 16 (21.1%) in 2018, and 21 (27.6%) in 2019. In accordance with the inquiry data, all of the 39 patients in 2017 were indigenous, while the remaining patients in 2018 and 2019 were imported cases. The detailed geographical distribution of clinical cases has been summarized in Table 1.

**Table 1:** Distribution of countries of origin for imported dengue cases.

Country	Years					
	2017		2018		2019	
	Indigenous cases	Imported cases	Indigenous cases	Imported cases	Indigenous cases	Imported cases
Maldives	\	\	\	4	\	\
Thailand	\	\	\	2	\	4
Cambodia	\	\	\	3	\	3
Malaysia	\	\	\	2	\	1
Vietnam	\	\	\	4	\	4
Singapore	\	\	\	1	\	2
Other countries	\	\	\	\	\	5
Total	37	0	0	16	0	21

### Phylogenetic analysis

**Figure 1:**
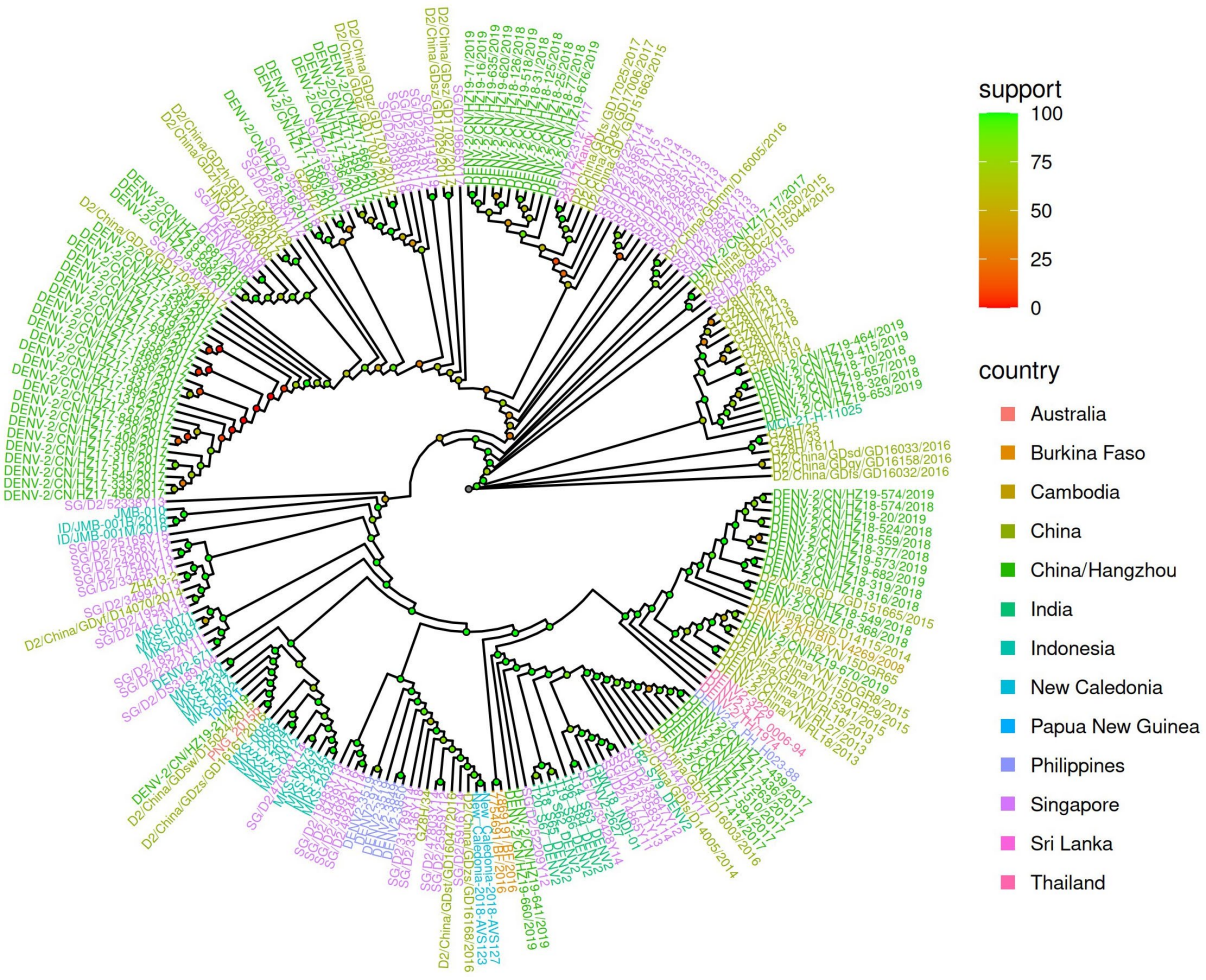
Phylogenetic analysis of 74 DENV-2 Cosmopolitan genotype genomes. The strains from 2017, 2018, and 2019 are labeled in red, blue, and orange lines, respectively. The inference sequences are marked in green lines. Node color marked as support represents bootstrap values of a phylogenetic analysis by IQ-Tree.

The phylogenetic analysis of 74 strains was carried out by aligning these viruses with 116 other representative DENV-2 viruses of diverse geographical origins obtained from GenBank (Figure 1). The findings indicated that 64.1% of the viruses in 2017 clustered in the same branch, close to strains that originated from Guangzhou-2017 (MN018361), Guangzhou-2017 (MH827547), and Singapore-2011 (MW512475). The MN018361 and MH827547 strains originated from Guangzhou and were imported from Malaysia. In 2017, other isolates were split into two branches and were close to Guangzhou-2017 (MH827552) and Guangzhou-2017 (MH827535), respectively, with both strains from Thailand. However, sequences from 2018 and 2019 were relatively close and mainly clustered into three branches, with the closest relative being Guangzhou-2015 (MH827537), Guangzhou-2018 (MK783191), Guangzhou-2018 (MK783192), and Guangzhou-2017 (MH827538).

### Amino acid mutation analysis of coding sequences

**Figure 2:**
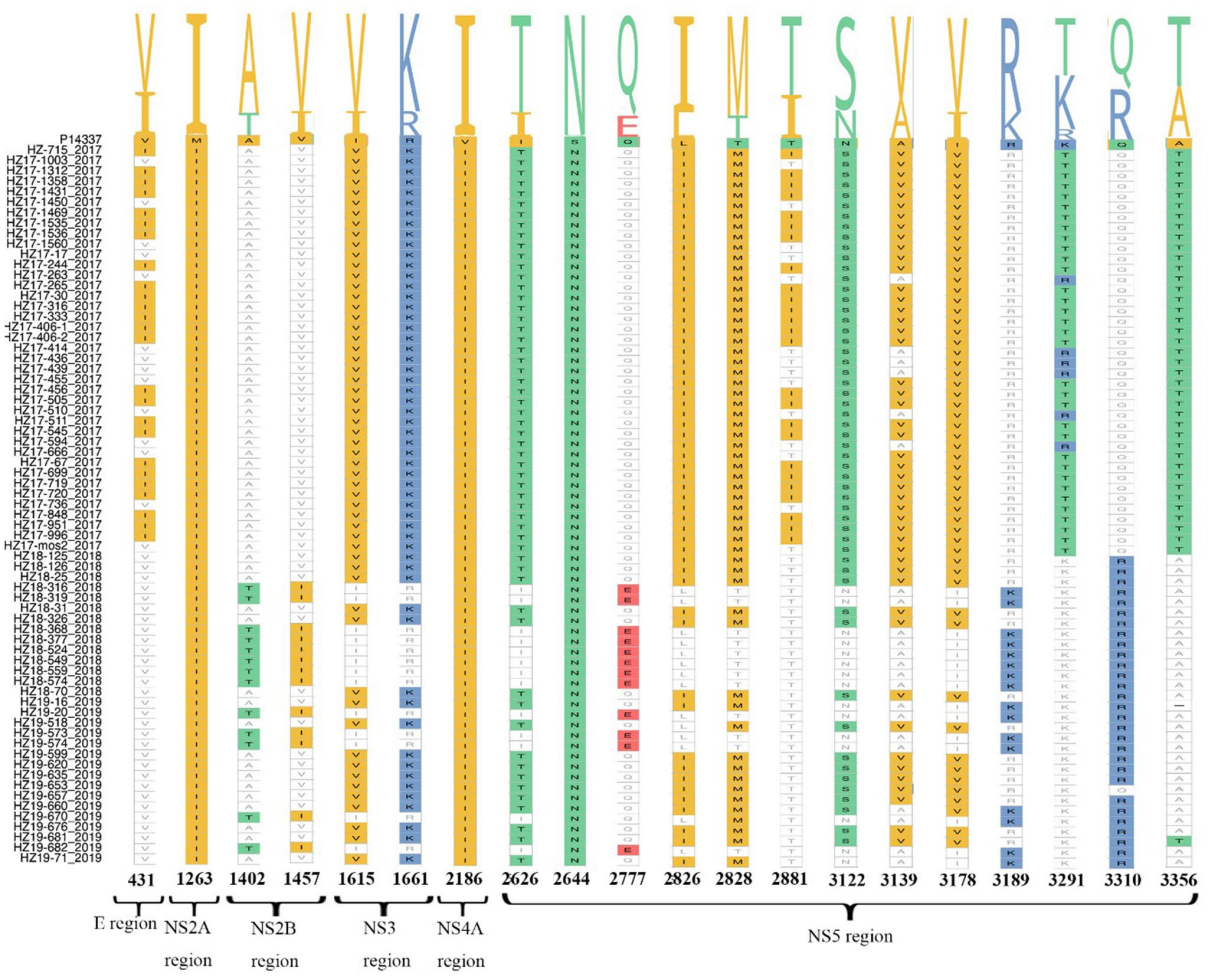
Amino acid substitutions in 74 complete genomic sequences of DENV-2. The amino acid mutations are labeled with different colors. The indicated numbers represent the amino acid change per position.

Compared to the reference strain P14337 (Thailand/0168/1979) in our study, a total of 20 specific amino acid (AA) mutations were identified, illustrated in Figure 2. The NS5 region presented the highest occurrence of AA mutations (13/20), while the NS4B region displayed the most conserved sequence with minimal AA mutations. V431I, T2881I, and K3291T mutations were identified solely in indigenous cases from 2017, in contrast to A1402T, V1457I, Q2777E, R3189K, and Q3310R mutations, which were exclusive to imported cases from 2018 and 2019. All 74 DENV-2 strains had the same M1263I, V2186I, and S2644N mutations.

### Evolutionary relationships of DENV-2 haplotypes

**Figure 3:**
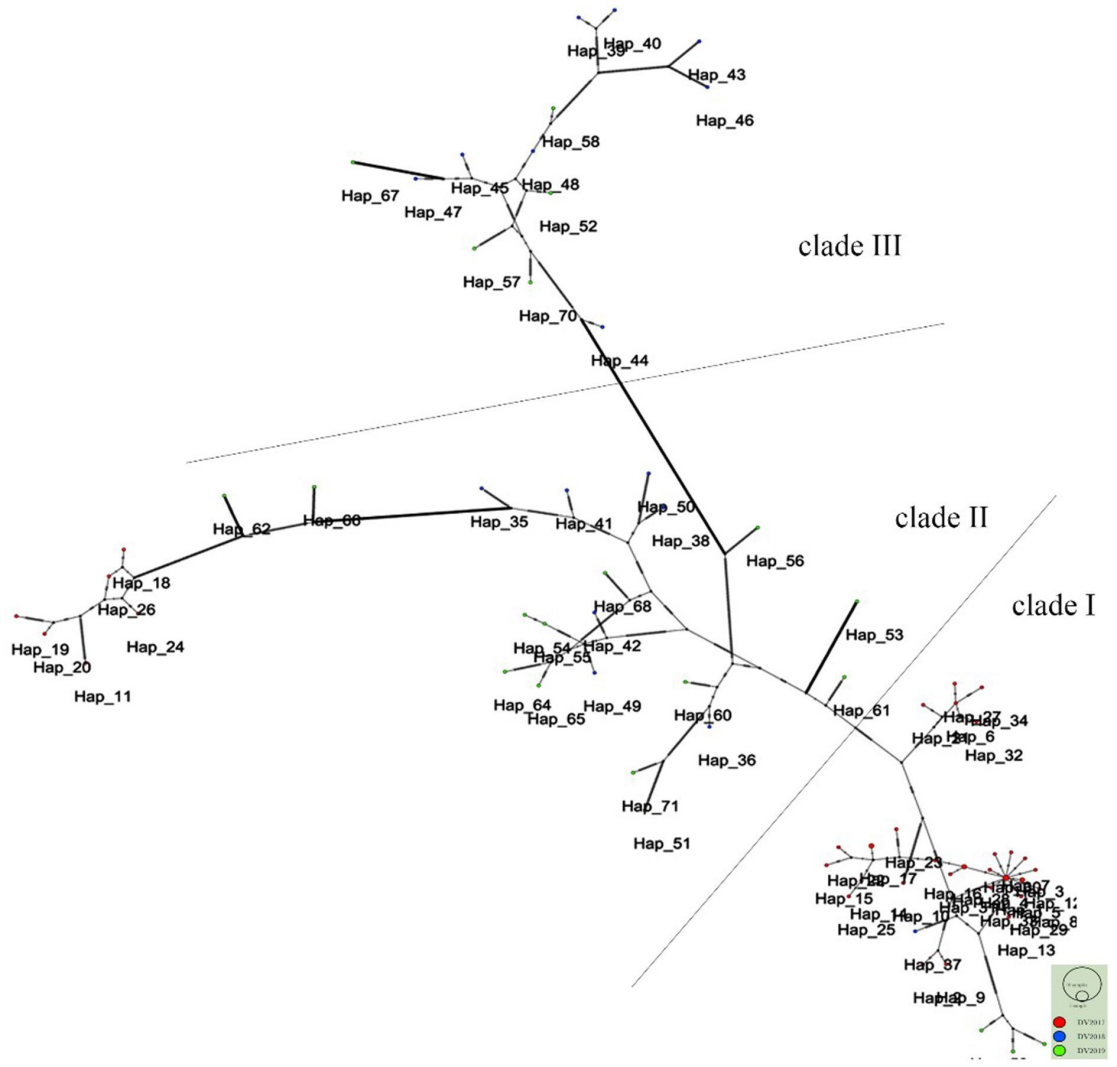
Phylogenetic networks of 74 DENV-2 strains. Each node in the network represents a haplotype. The red nodes represent the strains from 2017, and the blue and green nodes represent the strains from 2018 and 2019, respectively.

The study revealed that 74 haplotypes were classified into three main clades, as illustrated in Figure 3. Clade I comprised 37 haplotypes, Clade II, encompassed 26 haplotypes, and Clade III contained 13 haplotypes. A considerable 84.6% of haplotypes (33/39) in 2017 were identified as part of Clade I, whereas in 2018 and 2019, most haplotypes belonged to Clades II and III. Moreover, Clade III and some Clade II haplotypes with longer branches showed more mutations.

### Recombination analysis

**Figure 4:**
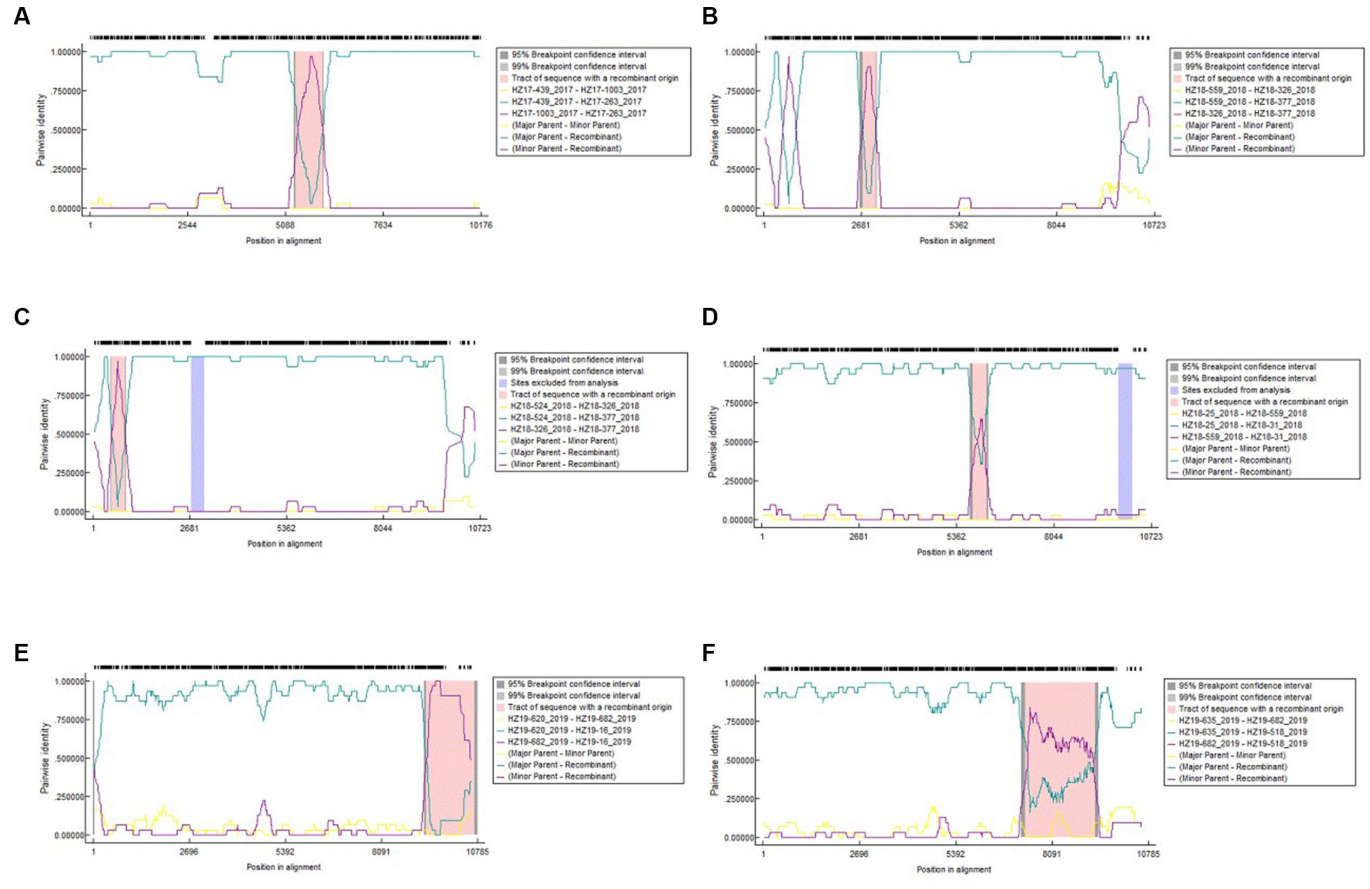
Analysis of recombination for 74 DENV-2 complete genomic sequences by RDP5. Each analysis considers different parent sequences (different colors) plotted in a graph assuming the vertical-axis pairwise identity and, on the horizontal axis, position on the genome. **(A)** The homology between genomes of HZ17-439_2017, HZ17-1003_2017 and HZ17-377_2017; **(B)** The homology between genomes of HZ18-559_2018, HZ18-326_2018 and HZ18-377_2018; **(C)** The homology between genomes of HZ18-524_2018, HZ18-326_2018 and HZ18-377_2018; **(D)** The homology between genomes of HZ18-25_2018, HZ18-559_2018 and HZ18-31_2018; **(E)** The homology between genomes of HZ19-620_2019, HZ19-682_2019 and HZ19-16_2019; **(F)** The homology between genomes of HZ19-635_2019, HZ19-682_2019 and HZ19-518_2019.

The result of recombination analysis indicated a total of 14 recombination events in the samples of 74 DENV-2 strains, with three occurring within strains isolated in 2017 and five and six detected within strains isolated in 2018 and 2019, respectively (Figure 4).

## Discussion

Dengue fever has been observed in more than 100 countries and regions, with over 50 million infections occurring annually (Poole-Smith et al., 2015). In recent decades, the incidences of dengue fever have increased in China due to various factors, such as urbanization, globalization, climate change, and migration (Brady et al., 2012; Murray et al., 2013; Li et al., 2018). Zhejiang province is located in the subtropical monsoon climate zone, characterized by warm and humid weather, which may lead to an epidemic of DENV (Wu et al., 2022). In 2017, an unforeseen epidemic in Zhejiang infected 1,229 individuals, where DENV-2 was the predominant serotype (Yan et al., 2018). Furthermore, there has been an increase in dengue fever cases in neighboring countries of China, such as Indonesia, Myanmar, Thailand, Singapore, and Malaysia, in recent years (Ng et al., 2015).

The genomic sequence of DENV-2 is essential for understanding the spread of dengue fever in Zhejiang Province. This study aimed to compare the genetic relationship between circulating DENV-2 viruses in Hangzhou and other regions worldwide through genomic phylogeny. We collected 74 DENV-2 strains from Hangzhou DF patients for the study, including 39 strains (51.3%) from 2017, 16 strains (21%) from 2018, and 21 strains (27.6%) from 2019. Hangzhou offers ideal conditions for the mosquito life cycle, including rivers, mountains, hills, and a temperate climate. Mosquito bites transmit DENV, and its infection rate is highly related to mosquito density. The spread of DENV infections is facilitated by population movement and trade that benefit from favorable geographic locations and frequent commercial exchanges. According to our data (Figure 1), multiple cases from other regions contributed to the outbreak and local epidemics in Hangzhou.

In contrast to previous studies by Yan et al. (2018) and Yao et al. (2021), the E gene was employed to analyze its phylogenetic, molecular, and epidemiological properties. Our research employed phylogenetic analysis and sequence alignment of the full-length genomes of strains collected in 2017, which showed a close relationship with isolates from Singapore, Malaysia, and Thailand. These findings suggest that the 2017 DENV-2 epidemic in Hangzhou originated in Southeast Asian countries like Malaysia, Singapore, or Thailand and then transmitted locally. Imported strains from 2018 were clustered with strains from 2019 and had close genetic relationships with Guangzhou strains, indicating possible importation from the same regions as those in Guangzhou. The genomic locations and specific strains associated with each recombination event were further examined. The results of this study provide important insights into the occurrence and frequency of recombination events in DENV-2 and illuminate novel avenues for further investigation.

Regarding our analysis of recovered strains, a comparison with the reference strain P14337 revealed 20 significant AA substitutions. Most of these substitutions (19/20) occurred in non-structural proteins, while only one mutation was observed in a structural protein; 65% (13/20) of the substitutions were in the NS5 region. Non-structural proteins play a crucial role in the life cycle and host immune suppression of DENV, and NS5 encodes the RNA-dependent RNA polymerase of the virus (Guan et al., 2021).

Mutations in NS5 may affect viral gene transcription or replication, enhancing virulence, and pathogenicity (Peng et al., 2022). The AA mutation analysis also showed that specific substitutions, such as V431I, T2881I, and K3291T, were present only in strains isolated in 2017. The other mutations, such as A1402T, V1457I, Q2777E, R3189K, and Q3310R, were exclusively found in the 2018 and 2019 cases. These results suggest distinct AA profiles between viruses isolated from indigenous cases in Hangzhou from 2017 and imported cases from 2018 to 2019. The impact of these amino acid mutation sites on the virulence and adaptability of the virus is yet to be explored.

In conclusion, this study performed the genetic analysis of the complete genome sequences of 74 DENV-2 from Hangzhou from 2017 to 2019. The new findings highlight that indigenous cases in 2017 had close genetic relationships with strains from Southeast Asian countries in the same year, while imported cases in 2018 and 2019 were phylogenetically linked with Guangzhou strains. This study may improve the prevention and control of cross-border transmission of DENV and provide further insight into DENV-2 transmission and local vaccine choice.

## Data availability statement

The datasets presented in this study can be found in online repositories. The names of the repository/repositories and accession number(s) can be found in the article/Supplementary material.

## Author contributions

HS: data analysis and writing the manuscript. WY: methodology and writing the manuscript. AS: data analysis and review the manuscript. FH: conceptualization and data analysis. MY: supervision and review the manuscript. All authors contributed to the article and approved the submitted version.

## Funding

This work was supported by the Zhejiang Provincial Key R&D Program of China (2023C03045 and 2022C03188) and National Health Commission Scientific Research Projects (WKJ-ZJ-2113 and WKJ-ZJ-2220).

## Conflict of interest

The authors declare that the research was conducted in the absence of any commercial or financial relationships that could be construed as a potential conflict of interest.

## Publisher’s note

All claims expressed in this article are solely those of the authors and do not necessarily represent those of their affiliated organizations, or those of the publisher, the editors and the reviewers. Any product that may be evaluated in this article, or claim that may be made by its manufacturer, is not guaranteed or endorsed by the publisher.
